# Quantitative Comparison of Photothermal Heat Generation between Gold Nanospheres and Nanorods

**DOI:** 10.1038/srep29836

**Published:** 2016-07-21

**Authors:** Zhenpeng Qin, Yiru Wang, Jaona Randrianalisoa, Vahid Raeesi, Warren C. W. Chan, Wojciech Lipiński, John C. Bischof

**Affiliations:** 1 Department of Mechanical Engineering, University of Minnesota, Minneapolis, MN 55455, USA; 2 Groupe de Recherche en Sciences pour l’Ingénieur (GRESPI) - EA 4694, University of Reims Champagne-Ardenne, 51687 Reims Cedex 2, France; 3 Department of Materials Science and Engineering, University of Toronto, Toronto, Ontario M5S 3G9, Canada; 4 Institute of Biomaterials and Biomedical Engineering, Department of Chemistry, Department of Chemical Engineering, University of Toronto, Toronto, Ontario M5S 3G9, Canada; 5 Donnelly Center for Cellular and Biomolecular Research, University of Toronto, Toronto, Ontario M5S 3E1, Canada; 6 Research School of Engineering, The Australian National University, Canberra, ACT 2601, Australia; 7 Department of Biomedical Engineering, University of Minnesota, Minneapolis, MN 55455, USA

## Abstract

Gold nanoparticles (GNPs) are widely used for biomedical applications due to unique optical properties, established synthesis methods, and biological compatibility. Despite important applications of plasmonic heating in thermal therapy, imaging, and diagnostics, the lack of quantification in heat generation leads to difficulties in comparing the heating capability for new plasmonic nanostructures and predicting the therapeutic and diagnostic outcome. This study quantifies GNP heat generation by experimental measurements and theoretical predictions for gold nanospheres (GNS) and nanorods (GNR). Interestingly, the results show a GNP-type dependent agreement between experiment and theory. The measured heat generation of GNS matches well with theory, while the measured heat generation of GNR is only 30% of that predicted theoretically at peak absorption. This then leads to a surprising finding that the polydispersity, the deviation of nanoparticle size and shape from nominal value, significantly influences GNR heat generation (>70% reduction), while having a limited effect for GNS (<10% change). This work demonstrates that polydispersity is an important metric in quantitatively predicting plasmonic heat generation and provides a validated framework to quantitatively compare the heating capabilities between gold and other plasmonic nanostructures.

Advances in material synthesis have produced a library of plasmonic nanomaterials with varying size, shape and composition^1^,^2^,^3^,^4^. These nanostructures are used in many biomedical applications including disease diagnosis^5^,^6^,^7^ and treatment based on their optical properties^8^,^9^. For instance, in diagnostics, nanoparticles have been used for visual labels for colorimetric bioassays including aggregation assays^10^ and lateral flow dipstick tests^11^. For therapeutics, nanomaterials have been studied to serve as drug nano-carriers^12^, and photothermal agents for tumor ablation once delivered to the tumor^13^. For all of these applications, it is increasingly important to quantitatively understand the effects of nanoparticle size, shape and composition to ensure reproducible biosensing and effective therapies.

For instance, given a nanomaterial, the size and shape determine the optical properties and interactions with biological systems. In the case of gold nanospheres (GNS), the plasmon resonance and hence color can be tuned within the visible spectrum from clear pink to dark red by changing the diameter (30–100 nm), and in the case of gold nanorods (GNR), the plasmon resonance can be tuned in the visible to near-infrared (NIR) spectrum from 600 nm to 1400 nm by changing their aspect ratio^3^,^14^. The biological responses, including cellular uptake^15^, internationalization pathway^16^, peri-vascular distribution in tumor^17^ and cytotoxicity^18^, also demonstrate a size- and shape-dependent behavior in recent studies. Although there have been both theoretical^19^,^20^,^21^,^22^,^23^ and experimental^24^,^25^,^26^,^27^ approaches to quantitatively account for photothermal heat generation from plasmonic nanostructures, there are no studies that integrate the experimental and theoretical approaches to quantify the heat generation, often leading to discrepancies^28^. This in turns leads to difficulties in predicting the therapeutic and diagnostic outcome and comparing the heat generating capability for new plasmonic nanostructures in biomedical applications.

In this study, we quantitatively measure the heat generation for GNPs, and examine the validity and conditions of agreement with theoretical predictions for the same nanostructures. Surprisingly, we showed that the agreement between experiment and theory is dependent on the type of GNP studied. Specifically, we found that the heat generation of GNS matches well with theoretical prediction, while the measured heat generation for GNR deviates significantly from theoretical prediction. This then led to an interesting finding that the polydispersity, i.e. the deviation of nanoparticle size and shape from nominal (i.e. average) value, significantly influences the optical properties of GNR including heat generation, but has limited influence on GNS. We further demonstrated the importance of accounting for the polydispersity by comparing the photothermal absorption for GNS and GNR with similar volume, leading to more realistic predictions. This work highlights the significance of polydispersity in determining the plasmonic nanoparticle heat generation and provides a framework to quantitatively compare the heating capability between plasmonic nanostructures.

## Results

### Dielectric constants and validation of discrete dipole approximation (DDA) with Mie theory

First, we calculated the size-dependent dielectric constants for GNS and GNR by correcting the bulk values measured by Johnson and Christy^29^. As shown in supplemental Figure S1, the real part of the dielectric constant does not change significantly with size while the imaginary part changes dramatically, especially in the near infrared (NIR) domain. The size effect is only significant for dimension smaller than 20 nm. Thus, the size dependent dielectric properties were only used if the particle is smaller than 20 nm (at least one dimension). To validate and establish DDA simulation protocol, we compared the results from DDA with Mie theory for a number of different sized gold nanoparticles (10 to 100 nm) and found good agreement between the two methods. Supplemental Figure S2(A,B) illustrates this comparison for a particle of 30 nm diameter.

### Comparing measured and predicted optical extinction and absorption for GNS

**Figure 1:**
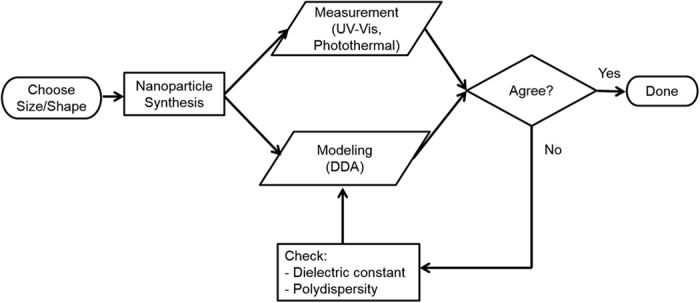
Flow-chart for the combined theoretical and experimental approach.

**Figure 2:**
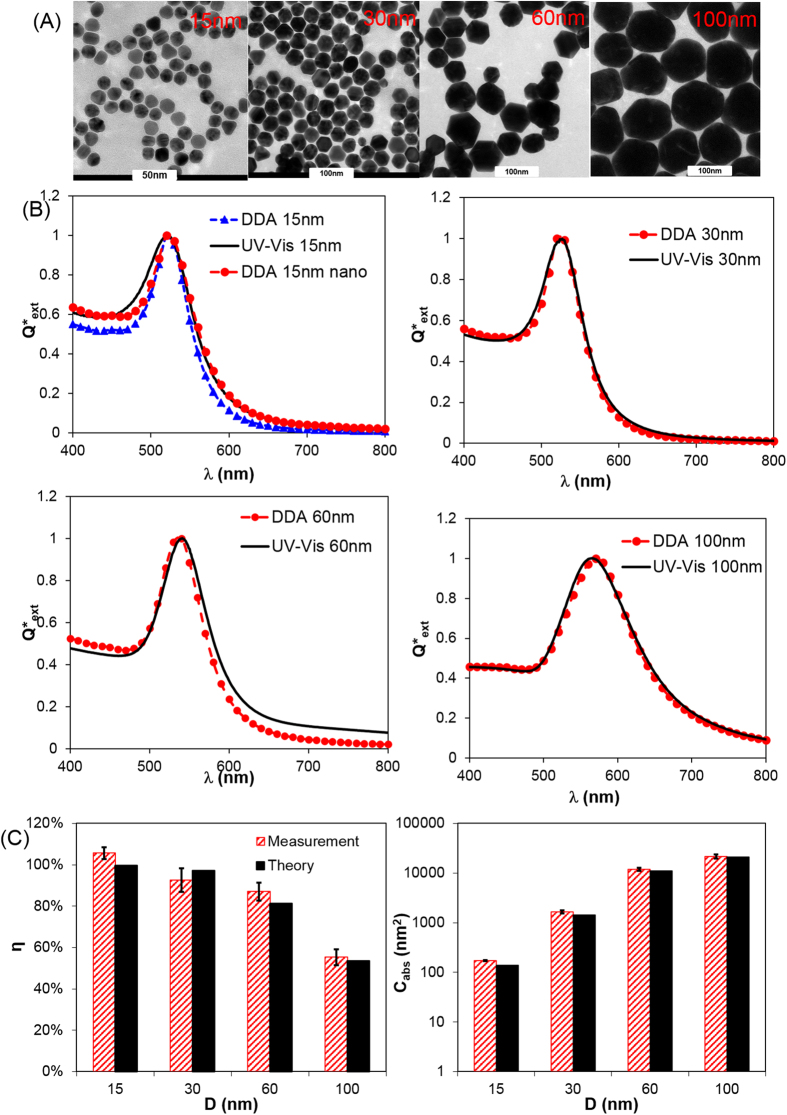
Comparison of DDA computation with experiment (i.e. UV–Vis spectroscopy and photothermal measurement) for GNS. (**A**) TEM images of 15 nm, 30 nm, 60 nm and 100 nm GNS; (**B**) Measured (UV–Vis) versus DDA-computed optical extinction spectrum; (**C**) Photothermal conversion efficiency (η) and absorption cross section (*C*_abs_ at 532 nm): quantitative measurement versus DDA prediction.

Next, we evaluated the agreement between DDA-predicted and experimentally measured optical properties for GNS with different sizes based on the flowchart shown in Fig. 1. As a first estimate, the mean diameter of the GNS was used to calculate the optical properties and good agreement with experimental measurement was observed as shown in Fig. 2. Here we systematically compared the measurement and theoretical prediction for the optical extinction spectrum (UV–Vis and Equation 8 in *Experimental Section*), photothermal absorption efficiency (Equations 1 and 9) and absorption cross section (Equation 2 and 8, directly related to heat generation). For 15 nm GNS, the measured plasmon peak is broader than DDA prediction. The size-dependent dielectric constants (referred to as DDA nano in Fig. 2) lead to a broader plasmon peak when compared with using the bulk dielectric constant, and a better agreement between DDA and experiment.

### Comparing measured and predicted optical extinction and absorption GNR

The calculation of the optical properties for GNR is more involved than for GNS due to the asymmetry. For instance with GNS, only one particle orientation is needed due to the symmetry. However for GNR, we suggest averaging two incident light polarization directions and different GNR orientations which are defined by two angles θ and ϕ with respect to the wave vector direction (Supplemental Figure S6A). Our calculations suggest with up to nine orientation directions of GNR with respect to the angle θ (defined as the angle between the rod longitudinal axis and the *x*-axis, Supplemental Figure S6A) is needed to reduce the error to be within 1% (Supplemental Figure S6B). We note our result differs from previous calculations suggesting averaging two directions is sufficient (along GNR long and short axis)^30^. In terms of dipole discretization, our calculation suggests 4 dipoles per nm gives an error within 1~2% over the entire considered wavelength interval (400–1000 nm, Supplemental Figure S7). Previous studies have used different dipole numbers ranging from 10^4^~10^7^ dipoles^30^,^31^,^32^ or a dipole spacing of 1 nm^20^. Sufficient amount of dipoles are needed for accurate prediction of nanoparticle optical properties and are manifested in two ways. First, adequate dipoles are needed to closely model the geometry of the nanoparticle. Second, the surface-to-volume ratio of dipoles is an important factor since excessive dipoles on the surface lead to overestimation of the absorption efficiency^21^,^31^,^33^.

**Figure 3:**
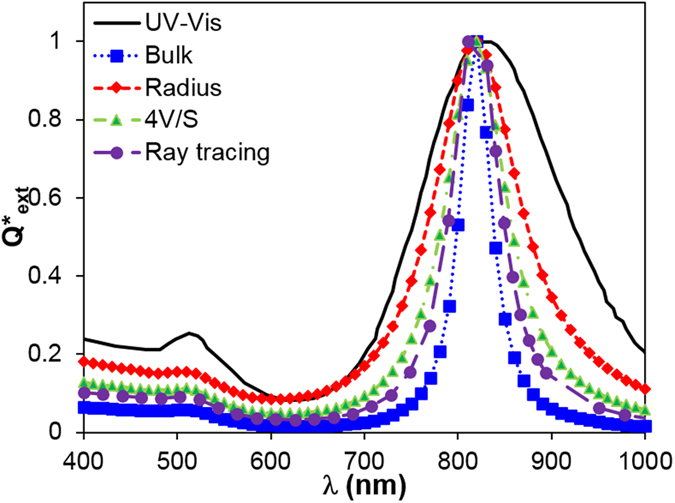
Predicted normalized efficiency factor Q^*^ of monodispersed GNR with nominal size, demonstrating a deviation from experimentally measured values. Dielectric constants include bulk and size dependent properties based on radius, 4*V/S*, and ray-tracing which considers anisotropy of GNR. Nanorod nominal size: *D* = 10.6 nm, *L* = 40 nm.

Next, we attempted to reconcile the optical properties of GNR between measurement and prediction. Unlike GNS, the UV–Vis measured optical spectrum does not agree well with DDA prediction with the nominal size of GNR as shown in Fig. 3. Specifically, the measured longitudinal peak (in the NIR region, wavelength range 700−900 nm) is much wider than that theoretically predicted, and the peak position is red-shifted (i.e., to longer wavelength) relative to the prediction. We further explored the effect of dielectric constant, including the bulk values and size-dependent properties using the radius of the rod (*D/2*)^30^, the effective size (equal to 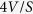), and anisotropic properties from ray-tracing calculation as shown in Fig. 3. Size-dependent properties lead to broader plasmon peak and lower extinction coefficients, with the radius-modified dielectric constant giving the broadest and lowest peak. However, the size-dependent and anisotropic properties do not explain the differences between experiment and prediction including the broadening and red-shift of the measured spectrum.

**Figure 4:**
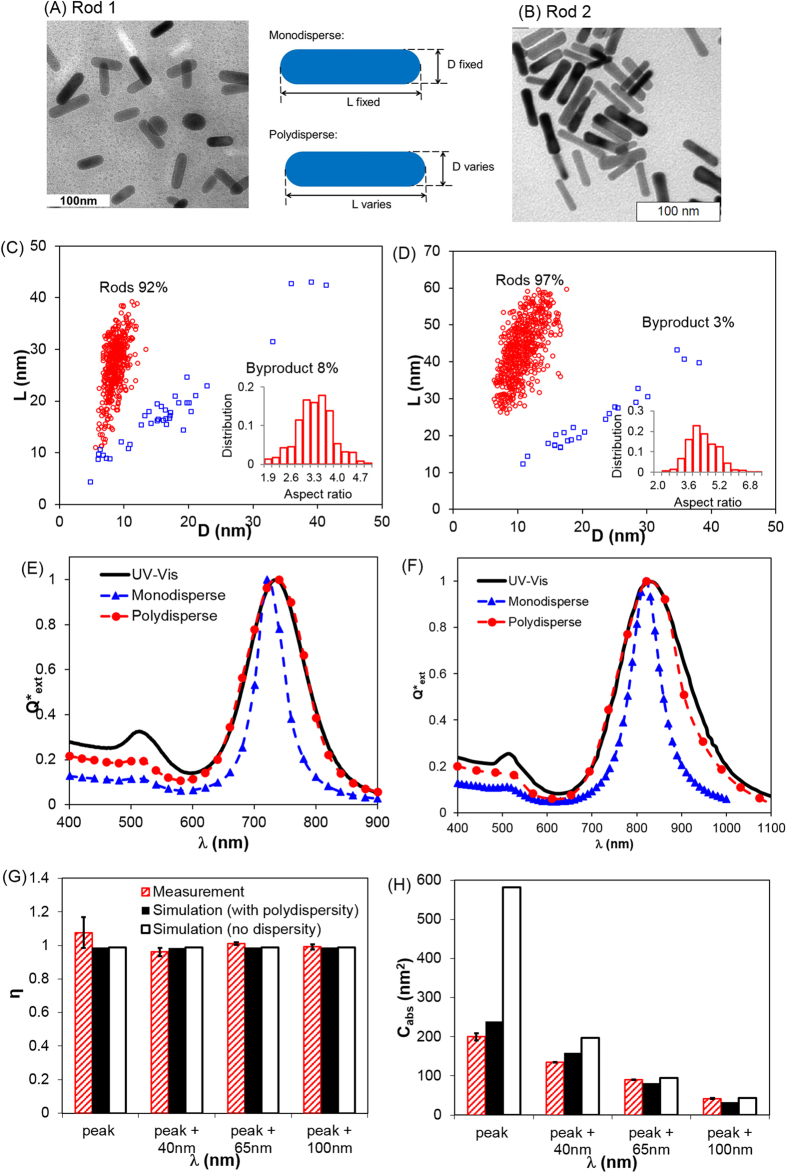
Inclusion of polydispersity into prediction leads to agreement between DDA prediction and UV–Vis measurement for GNR. Two GNR were studied with nominal sizes (**A**) *D* = 10.6 nm and *L* = 40 nm; (**B**) *D* = 8.6 nm and *L* = 27 nm; (**C,D**) Polydispersity measurement. The polydispersity data for Rod 2 was reproduced from Khlebstov *et al*. with permission^34^. (**E,F**) Measured (UV–Vis) versus DDA-computed optical extinction spectrum; (**G,H**) Photothermal conversion efficiency (η) and absorption cross section (*C*_abs_): quantitative measurement versus DDA prediction for Rod 1 (peak refers to 740 nm).

Subsequently, we examined the polydispersity (i.e. size and shape distribution) of GNR and attempted to incorporate this variable into optical properties prediction. Through detailed TEM image analysis, the distribution of the length and diameter of GNR can be obtained (Fig. 4A,C). There are both GNR (with varying diameter and length), and “byproducts” which mostly consist of spheres and cubes, in accordance with earlier reports^34^. We further showed that the aspect ratio of GNR can be approximated by a Gaussian distribution (*Supplemental Information* section 2, Table S1 and Figure S4). We then calculated the optical properties (*C*_abs,_ *C*_sca_) of GNR using the nominal diameter (D) and length values (L) within one standard deviation (σ) of the mean (μ) of the Gaussian distribution. By properly weighting the optical properties according to their percentage distribution (Table S1 and Equation 11), this led to a satisfactory agreement between experiment and theory for GNR as shown in Fig. 4E,G,H including UV–Vis optical spectrum and quantitative measurement of photothermal conversion efficiency and absorption cross section. To further check the applicability of this approach, we reproduced the polydispersity data of a different nanorod (D = 10 nm, L = 40 nm) from Khlebtsov *et al*.^34^ and obtained similar agreement when taking the polydispersity into account (Rod 2 in Fig. 4B,D).

**Figure 5:**
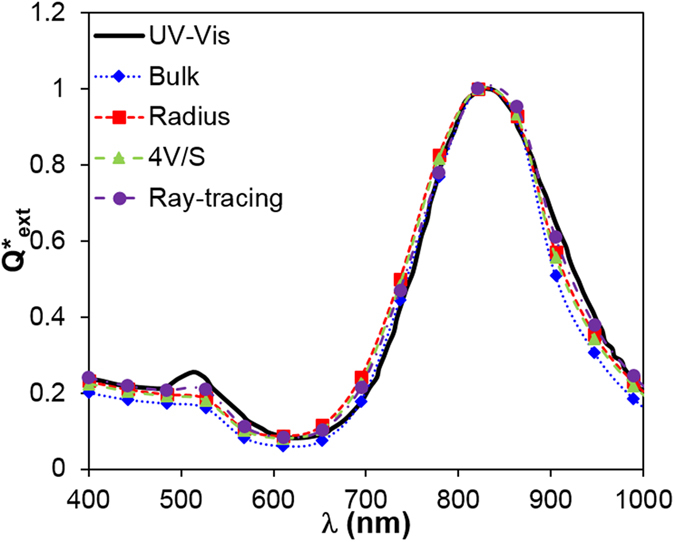
Dielectric constants do not significantly change the optical properties of GNR (*D* = 8.6 nm, *L* = 27 nm) after incorporating polydispersity measured from TEM (shown in Fig. 4).

Furthermore, we tested the sensitivity of optical properties to size-dependent dielectric constants after incorporating polydispersity. Interestingly, the optical extinction spectrum does not change significantly when using size-dependent dielectric constants, as shown in Fig. 5. The bulk and size-dependent dielectric constants (with radius, 4V/S and ray-tracing) all give similar predictions and agree well with the UV–Vis measurement.

### Effects of polydispersity on GNS and GNR

**Figure 6:**
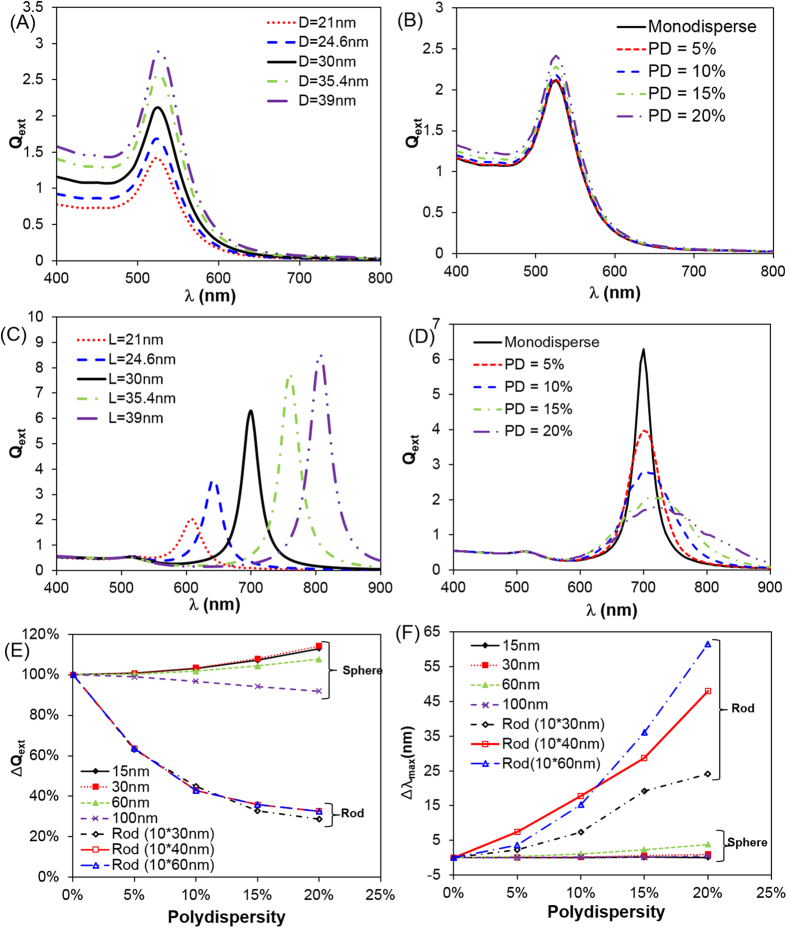
Differential impact of polydispersity for GNS and GNR. (**A,B**) Changing polydispersity does not significantly affect the optical properties of GNS (*D* = 30 nm in this example); (**C,D**) polydispersity changes the optical properties of GNR including extinction peak and peak width. Polydispersity is modeled by fixing *D* = 10 nm and varying *L*; (**E**) Summary of the impact of polydispersity on the optical properties (peak extinction efficiency) of GNS and GNR. Here polydispersity is defined as the standard deviation by the mean. (**F**) Impact of polydispersity on peak wavelength shift.

Lastly, we examined the impact of polydispersity on the predicted optical properties for GNS and GNR. Here polydispersity is quantitatively defined as the ratio of standard deviation to the mean value (*σ*/*μ*), assuming a Gaussian distribution. As shown in Fig. 6, the polydispersity leads to less than 10% change in the extinction peak and less than 5 nm shift in the resonant wavelength for GNS. In contrast for GNR, the same polydispersity range leads to more than 70% reduction in extinction peak, broader spectrum, and more than 20 nm red-shifts in the resonant wavelength. The differential impact of polydispersity originates from the sensitivity of the nanostructure to size and shape variation and has important implications in nanostructure design particularly for optical applications. For GNS, the spectrum and plasmon peak are less sensitive to the size change, and the spectrum tends to compensate for each other around the nominal size (Fig. 6). For GNR, however, the optical extinction spectrum and plasmon peak are highly sensitive to the change in size and aspect ratio.

## Discussion

In this study, polydispersity was obtained by imaging GNR under TEM and analyzing the distribution of the size parameters (diameter and length). While the two GNR compared in this study have 3% and 8% byproducts, the percentage of the byproducts can go up to 10 to 20% as reported previously^35^,^36^. These byproducts, for instance cubes if in large quantities (not observed in this study), can lead to new peaks in the optical extinction spectrum^35^. The byproducts typically have plasmon resonance in the visible range (500–600 nm) with limited interaction in the near infrared range; however, they will lead to lower absorption of laser energy in the near infrared range if considering molar heating or heat generation per Au mass.

Comparing the heat generation from theoretical prediction and experimental measurement requires accuracy in both approaches. The accuracy of DDA prediction depends on the choice of the discretization. Smaller dipole spacing leads to more accurate results. From this study, over 250,000 dipoles are needed to generate results within 1% accuracy of Mie theory. Compared with Mie theory, DDA has the advantage that it can handle targets with complex geometry (i.e. nanorods). In addition, the substance constituting the target can be non-homogeneous and even with anisotropic properties. Furthermore, incorporating size-dependent properties leads to better agreement for small GNS (<20 nm, Figs 2B and S5), and also broader plasmon peak. The broadening of the plasmon peak due to the size-dependent dielectric constant (i.e. electron scattering with boundary) is in agreement with previous observations from both experiment and calculations in the literature^20^,^37^. In addition to the electron-boundary scattering, other factors can contribute to the size-dependent dielectric functions for small particles including chemical interface damping (CID) and quantum effects^38^. The mechanism of CID, i.e. the fast energy transfer between nanoparticle and its immediate environment, is not well understood and the experimental study is challenged with polydisperse nanoparticle distribution, both of which lead to plasmon damping and spectrum broadening^39^,^40^. CID is typically represented by the *A* value in Equation 1 and *A* = 1/3 is used in the literature for gold nanoparticles in water^34^,^41^. Quantum effect can take place for particles smaller than 10 nm^37^,^42^. For instance, we investigated a GNS with diameter 8.9 nm from NIST (NIST–RM8011, Supplemental Figure S5) and found that the measured plasmon peak is significantly broadened compared with DDA prediction with bulk properties or size-dependent properties. The details of how to account for the quantum effect are discussed elsewhere^37^.

Currently we are unaware of any standard method to quantitatively measure the bulk heat generation resulting from laser nanostructure interactions. Previous studies have reported a variety of different methods including laser heating in a water droplet^25^, a cuvette in vacuum (isolating heat convection losses^43^), and cuvette in standard room temperature and pressure^24^. The reported results vary significantly among different studies^25^,^43^ and a relative heat generation was frequently reported due to the lack of known heat generation^24^,^25^,^43^. Indirect measurement using photoacoustic imaging offers some insight into the photothermal absorption^28^. In this study, we have found that the heat generation calibration mitigates previous difficulties and is a key factor in obtaining accurate thermal measurement. Specifically, a known amount of energy by resistive heating was delivered using a small resistor in a cuvette^44^. The equilibrium temperature change was then linearly correlated with the energy input. This linear calibration curve was used to quantify the heat generation from laser GNP heating, leading to reproducible and accurate results within the test cuvette (Supplemental Figure S3).

After validating DDA-predicted optical properties with experimental measurements, we then systematically analyzed the impact of polydispersity on the optical properties of plasmonic nanoparticles. Here we define the polydispersity as the ratio of standard deviation to the mean of Gaussian distribution (*σ*/*μ*). For GNS, the mean and standard deviation values refer to the diameter; while for GNR, the values refer to the length or aspect ratio. While polydispersity has a limited effect on GNS (Fig. 6), it plays a dominate role in the optical properties of GNR and leads to broader spectrum and red-shift compared with the prediction using the nominal size. We attribute the strong polydispersity dependent GNR optical properties due to the large shift in the plasmon peak when varying the aspect ratio (Fig. 6C). However for GNS, the polydispersity leads to the nanoparticle size variation which mainly alters the magnitude of the optical properties but does not shift the plasmonic peaks significantly (Fig. 6A). More interestingly, the effect of size-dependent dielectric constants are insignificant when polydispersity of GNR was taken into account (Fig. 5). This is likely due to the fact that polydispersity already broadens the GNR plasmonic peak, thus the additional broadening from size-dependent dielectric constants does not leads to significant changes.

It is worth clarifying that the photothermal conversion efficiency does not represent the ability of the plasmonic nanoparticles to generate heat. Instead, the photothermal conversion efficiency describes how the nanoparticle disposes the incident electromagnetic energy, either by absorption or scattering. This is easily perceived from the definition of the photothermal efficiency as the ratio of the heat generation and the laser power loss from experiment (*μ*_measure_ = *Q*/*P*_laser_, where *Q* is the sample heat generation and *P*_*laser*_ is the laser power loss in the sample), or equivalently the ratio of absorption and extinction cross sections from prediction (*η*_theory_ = *C*_abs_/*C*_ext_). On the other hand, the heat generation capability of plasmonic nanoparticles is directly related to the absorption cross section, which can be determined by quantifying the nanoparticle heat generation (*C*_abs_ = *Q*/(*N* · *I* · *V*), where *N* is the number density of gold nanoparticles, *V* is the sample volume, and *I* is the laser intensity). For instance, increasing GNS size from 15 nm to 100 nm reduces the photothermal conversion efficiency from 100% to 54% (Fig. 2C), but a 100 nm GNS generates 150 times more heat than a 15 nm GNS. In general, increasing the nanoparticle size leads to enhanced scattering, reducing the photothermal conversion efficiency^24^,^26^,^27^, but does not indicate that less heat is generated on a per nanoparticle basis. A similar trend is expected for nanoparticle aggregates or assemblies, i.e. several small nanoparticles linking with each other. These nanoparticle ensembles act as larger nanoparticles with increased absorption cross section (per nanoparticle) leading to lower photothermal conversion efficiency due to increased scattering.

For the GNR studied, the photothermal conversion efficiency does not depend on wavelength (99%, Fig. 4G), however, the absorption cross section from GNR reduces by 7-fold by red-shifting 100 nm from absorption peak (Fig. 4H). It is also worth mentioning that the GNR undergoes melting and shape change when subjected to ultra-short (such as femtosecond or nanosecond) laser pulses^45^,^46^. The shape change will have a significant impact on the optical properties including photothermal heat generation and conversion efficiency. This study focuses on the nanoparticle heating under continuous wave laser with the highest bulk temperature below 50 °C, and did not observe significant change in the absorption peak before and after laser irradiation (Supplemental Figure S8). Furthermore, we focused on the behavior of individual GNR that is well separated from each other. When GNRs are linked close together to form dimers or more complex assemblies, the optical properties change dramatically due to the plasmon coupling and depend strongly on the relative position and orientation of individual particles within the assembly^47^,^48^,^49^,^50^.

**Figure 7:**
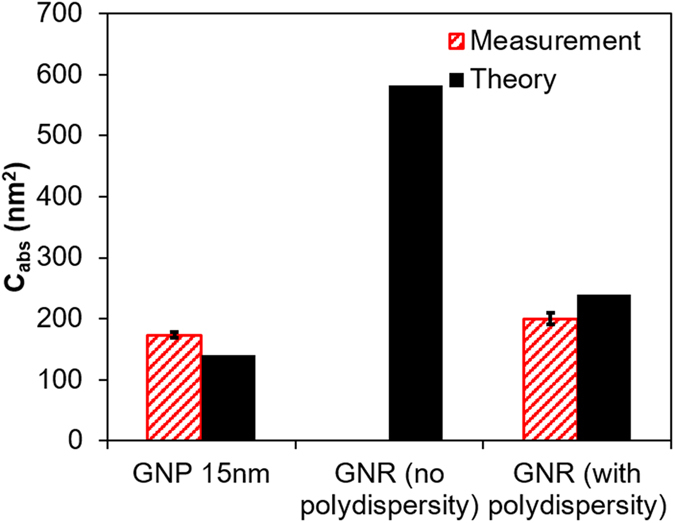
Incorporating polydispersity to predict plasmonic photothermal absorption and heat generation. The absorption cross section of a GNS (15 nm) is compared with a GNR (Rod 1 in Fig. 4 at peak absorption, with similar gold volume with 15 nm GNS) with and without accounting polydispersity.

This work has important implications especially for biomedical applications requiring an accurate estimation of heat generation from laser gold nanomaterial interactions such as photothermal therapy for cancer^13^ and thermal contrast biosensing diagnostics^51^. While previous studies have focused on matching the plasmon peak (*λ*_max_) between experiment and theory^30^,^52^, the magnitude of absorption, and thus the associated optical properties, are critical to determine the amount of heat generation. As shown in this work, the absorption efficiency of GNR can degrade significantly with polydispersity, in contrast to GNS. This is important since GNR are often advocated as more efficient light absorbers than GNS at their tuned plasmon resonances. For instance, it has been suggested that a GNR can absorb 3~5 times more light energy at the plasmon resonance than GNS with the same gold mass (i.e. only changing morphology)^22^; however, based on the present work this enhancement can diminish dramatically when taking into account the effect of polydispersity (ca. 10~20%, Fig. 7). Our study speaks to the need for reporting polydispersity along with size and shape in order to accurately estimate the optical properties and hence heating potential under laser irradiation for GNR. It can be anticipated that polydispersity will also play an important role in photothermal conversion for other increasingly complicated gold nanomaterials (such as shell, cube, stars, horns etc.)^13^,^53^.

## Conclusion

Plasmonic nanoparticle heating has been applied in thermal therapy, imaging, and diagnostics. However, the lack of quantification of the heat generation from plasmonic nanostructures has led to difficulties in predicting the therapeutic and diagnostic outcome of specific applications, and hinders our ability to compare the heat capabilities between new plasmonic nanostructures. In this study, we quantitatively compare the experimentally measured vs. predicted optical properties including heat generation of GNS and GNR. This revealed a surprising finding that polydispersity has a significantly different impact on the optical performance of plasmonic nanostructures. Specifically, changing the polydispersity (defined ratio of standard deviation to the mean of Gaussian distribution, *σ*/*μ* = 0~20%) leads to less than 10% change in optical extinction and absorption for GNS, while the same polydispersity range results in more than a 70% reduction for GNR. This work demonstrates the importance of reporting both polydispersity and nominal size and shape for plasmonic nanostructures. Further, it provides a framework to use this information to quantitatively determine and compare heating between increasingly complex plasmonic nanostructures in the future.

## Methods

Experimental and computational approaches to study GNP properties are discussed in this section. First, the synthesis and characterization of GNPs are discussed including the synthesis of GNS and GNR and laser heating measurement. Next, the computational framework for GNS optical properties is discussed including the input parameter (dielectric function) for the Mie theory and discrete dipole approximation (DDA). Mie theory is only established for simple geometries such as spheres and thus is used as an analytical benchmark for the DDA calculation which can handle complex geometries including the GNR.

### GNS and GNR synthesis and characterization

#### GNS Synthesis

GNS were synthesized according to established protocols with a modification of the Frens method^54^. Basically, 1% sodium citrate (Sigma-Aldrich, unless otherwise specified) was used to make 15 nm gold nanoparticles by boiling gold chloride. For larger GNS, hydroquinone reduction was used to synthesize 30 nm, 60 nm, and 100 nm particles. Particle stability was maintained by adding Tween 20 during centrifugation and purification. Gold nanoparticle reference materials (RMs) from the National Institute of Standards and Technology (NIST) were also compared including primary particle diameters of nominally 10 nm, 30 nm, and 60 nm (NIST RMs 8011, 8012 and 8013).

#### GNR Synthesis

GNR were synthesized with standard protocols in previous publications^55^ developed by the Murphy group^3^ and the Liz-Marzan group^56^. Briefly, gold seed precursor solution was prepared by adding 375 μL of 0.01 M chilled sodium borohydride to 0.9 mL of 0.1 M gold chloride solution in 14.625 mL of 0.1 M CTAB surfactant. A second precursor solution is made by combining 48 mL of 0.01 M gold chloride and 9.8 mL of 0.01 M silver nitrate into a 1 L Erlenmeyer flask containing 933 mL of rapidly stirring 0.1 M CTAB. GNR growth is then initiated by aliquoting 6.86 mL of 0.1 M ascorbic acid and 12 mL of gold seed precursor solution into the flask and stirring overnight.

#### GNS and GNR Characterization

The gold nanoparticles were characterized by UV–Vis spectroscopy (Synergy HT, BioTek) for extinction spectrum, and transmission electron microscopy (TEM, Tecnai G2, FEI, 120 kV) for size distribution. For TEM, a drop of gold nanoparticle solution was placed on a TEM grid for 15 min and then dried with filter paper. After acquiring the TEM images, the size parameters (diameter for GNS, diameter and length for GNR) were analyzed using imaging processing software Fiji (ImageJ with plugins). For NIST GNSs, the optical properties and size distributions data were obtained from NIST Report of Investigation documents.

#### Laser Heating to Measure GNP Photothermal Absorption

1 mL of gold nanoparticle solution was loaded in a polystyrene cuvette. The solution is heated with a beam of laser at varying wavelengths (532 nm for GNS, 700 to 850 nm for GNR, ~200 mW, Spectral-Physics Millennia Vs and 3900 S) from the side and the temperature is recorded by four T-type thermocouples located in the corner of the cuvette while a small magnetic stirrer is placed inside the cuvette to obtain a uniform temperature reading (Supplemental Figure S3). The solution is heated from room temperature to a steady temperature (i.e. balanced heat gain with loss to environment) which requires roughly 30 min to 45 min, and then allowed to cool down to room temperature (30 min). To accurately determine the amount of absorption and heat generation, we obtained a calibration curve by heating up a 100 Ω resistor with known voltage. The amount of heat generation shows a linear relationship with the temperature change between the steady temperature and room temperature (*Q* = 16.855Δ*T*, mW), a condition that heat generation equilibrates with heat loss to the environment. The amount of heat generation from the laser heating is then determined from this calibrated linear relationship. The laser power entering and exiting the sample is measured by a power meter to determine the total laser power loss (*P*_laser_). After accounting for the refractive index mismatch, the photothermal efficiency is calculated by


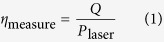


The measured absorption cross section of the nanoparticle is calculated by,


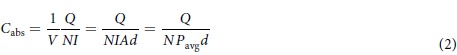


where *N* is the number density of gold nanoparticles (1/m^3^), *V* is the volume of the solution (1 mL), *I* is the laser intensity (W/m^2^), *P*_avg_ is the average laser power (W) at which the sample is irradiated, and *d* is the depth of the solution that laser travels through (i.e. 1 cm). Here the average laser power is taken in the form of logarithmic mean of the incident and transmitting laser power (*P*_avg_ = (*P*_in_−*P*_out_)/(ln *P*_in_−ln *P*_out_)) to accurately account for the exponentially decaying laser intensity in the sample.

### Computation of GNP Optical Properties

#### Dielectric Functions and Their Size & Directional Dependence

Complex dielectric functions (*ε*_bulk_) for gold from Johnson and Christy^29^ were used. Based on earlier investigations^42^, the size effect on dielectric functions can be captured by using a damping constant, γ(*L*_eff_), to account for electron scattering with particle boundary:


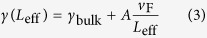


where *γ*_bulk_ is the electron collision frequency in bulk material, *v*_*F*_ is the Fermi velocity, *A* is a scattering parameter (~0.33^41^), and *L*_eff_ is the mean-free-path (or effective length) of electron-boundary scattering. Given that *L*_eff_ refers to the average geometrical path of electrons from surface to surface of the particle, it is known from other fields of physics that such mean geometric path can be given by “the mean-beam-length of a radiation bundle in gas filled cavity”^57^,


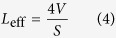


where *V* is the volume and *S* is the surface area of the GNPs. Equation 4 was first used by Coronado *et al*.^58^ to account for the size effect on dielectric function of spherical GNPs and leads to *L*_eff_ = 4*a*/3 with particle radius *a*. According to the Drude permittivity model, the size dependent dielectric function can be written as


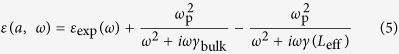


where *ε*_exp_(*ω*) is the dielectric function of bulk sample measured from experiment, *ω*_*p*_ is the bulk plasmon frequency of gold.

Note that Equation 4 corresponds to the direction averaged paths within the nanoparticles. For GNR, the surface scattering in the transverse (i.e. width D in Fig. 4) direction is intuitively expected to be more pronounced than that in the longitudinal (i.e. length L) direction, leading to a directionally dependent dielectric function. To estimate the surface scattering lengths in longitudinal and transverse directions and thus to quantify the effect of anisotropy on the dielectric function, we use a ray-tracing stochastic approach. In this approach, the effective lengths for transverse (*L*_eff, *D*_) and longitudinal (*L*_eff, *L*_) directions can be estimated by following relations respectively:


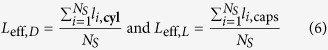


where *l*_i, cyl_ and *l*_i, caps_ are the path lengths of an electron “*i*” started from the cylindrical surface and end cap(s) to any other surface of the GNR respectively. *N*_*S*_ is the number of electron samples chosen as 10,000 here. The initial position and direction of each electron sample are chosen in a random manner as detailed elsewhere^59^,^60^.

#### Mie Theory Calculation

The Mie theory provides exact values of the far-field extinction, absorption and scattering efficiency and asymmetry factors for a spherical particle suspended in a non-absorbing host medium illuminated by an incident plane wave^61^


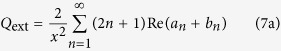



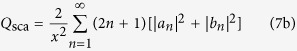



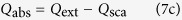



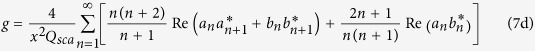



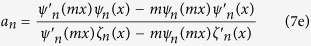



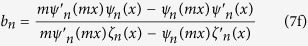


where *x* is the particle size parameter (=2π*a*/λ), *m* is the ratio of complex refractive index (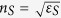) of the sphere to that of the surrounding medium (*n*_*m*_), *ψ*_*n*_ and *ζ*_*n*_ are spherical Bessel functions, and the asterisk (*) and prime ( ′ ) indicate complex conjugate and derivative with respect to the argument *x* or *mx*, respectively.

### Discrete Dipole Approximation (DDA)

Discrete Dipole Approximation (DDA) is a discrete solution method of the integral form of Maxwell’s equations and allows the prediction of nanostructure optical properties with complex geometries beyond Mie theory.^26^ Basically, the target structure is discretized into a finite array of dipoles (*N)* with each one located at position *r*_j_ (*j* = 1, *N*). After solving 3 N complex linear equations with unknown dipole moments^31^, the extinction, absorption and scattering cross sections and asymmetry factor can by calculated by


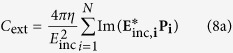



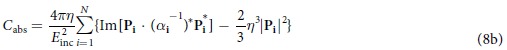



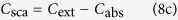



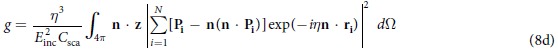


where **z** is the direction of the incident plane wave of amplitude *E*_inc_, **n** is a unit vector of the scattering direction, *d*Ω is the differential solid angle around of **n**, **E**_inc, **I**_ is the incident electric field vector on the dipole **i**, **P**_**i**_ is the dipole moment vector, * η* (=2π/λ) is the wave number, and *α*_*i*_ is the polarizability of the dipole **i**. The predicted photothermal conversion efficiency is defined as


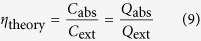


In this study, the DDA package DDSCAT 7.2 developed by Draine and Co-workers^31^ was implemented. To generate spherical particles and rods with hemispherical end caps, we use the DDSCAT predefined programs, which create the target objects as regular arrays of dipoles of spacing *d*. The discrete dipole spacing should be small as compared to any structural length in the target geometry, and the wavelength of the electromagnetic wave (λ). A convenient “rule of thumb” developed to satisfy these criteria is


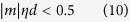


#### Orientation Averaged Optical Properties

For GNS, Equation 8 is valid for any orientation of the GNP with respect to the incident wave direction due to isotropy of spheres. However, these parameters have to be computed for various GNR orientations and then averaged since GNR are generally randomly oriented in an aqueous solution. DDSCAT code allows us to compute the cross sections in a set of directions and then determine the minimum number of directions for the orientation-averaged extinction, absorption, and scattering cross sections, and the orientation-averaged asymmetry factor.

#### Polydispersity

The nanoparticle size distribution was discretized into a number of bins (i.e. intervals, supplemental Table S1 and Figure S4) and then weight-averaged to obtain the ensemble optical properties.


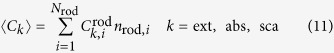


with *N*_rod_ the number of bins for rods according to TEM image analysis. *n*_rod,i_ = *N*_rod,*i*_/*N*_rod_ where *N*_rod, *i*_ is the number of rods whose sizes are inside the bin *i*; 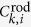
orientation-averaged *k* (i.e. extinction, absorption, or scattering) cross sections of a rod of size inside the bin *i*.

## Additional Information

**How to cite this article**: Qin, Z. *et al*. Quantitative Comparison of Photothermal Heat Generation between Gold Nanospheres and Nanorods. *Sci. Rep.* **6**, 29836; doi: 10.1038/srep29836 (2016).

## Supplementary Material

Supplementary Information
