# Supplementary material for: Quantitative Comparison of Photothermal Heat Generation between Gold Nanospheres and Nanorods
Source: Sci Rep. 2016 Jul 21;6:29836. doi: 10.1038/srep29836 (PMC4956767; doi:10.1038/srep29836)
Supplement: Supplementary Information [file srep29836-s1.pdf]

Supplementary Information

Quantitative Comparison of Photothermal Heat Generation between Gold Nanospheres and Nanorods

*Zhenpeng Qin, Yiru Wang, Jaona Randrianalisoa, Vahid Raeesi, Warren C. W. Chan, Wojciech Lipinski, John C. Bischof **

Table of Contents

1. Validation of DDA simulation 2

2. Photothermal heating experimental setup 3

3. Measured GNR size distribution 4

4. Comparison of DDA simulation and UV–Vis measurement for NIST particles. 5

5. DDA Calculation protocol for GNR 6

6. UV-Vis spectrophotometry for GNR before and after laser heating 8

1. Validation of DDA simulation

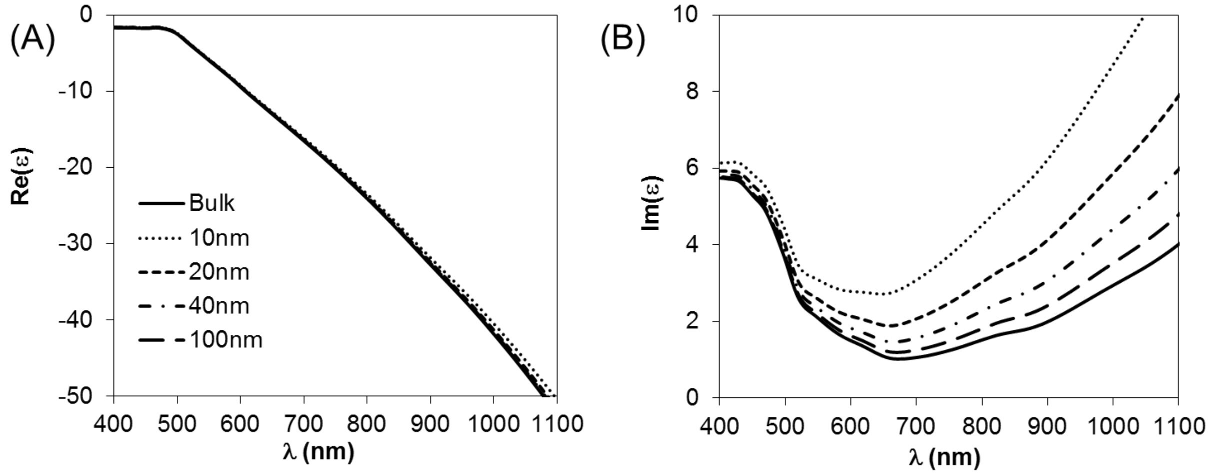

Figure S1. Dielectric constants for gold metal as a function of gold nanoparticle size. (A) Real part and (B) Imaginary part.

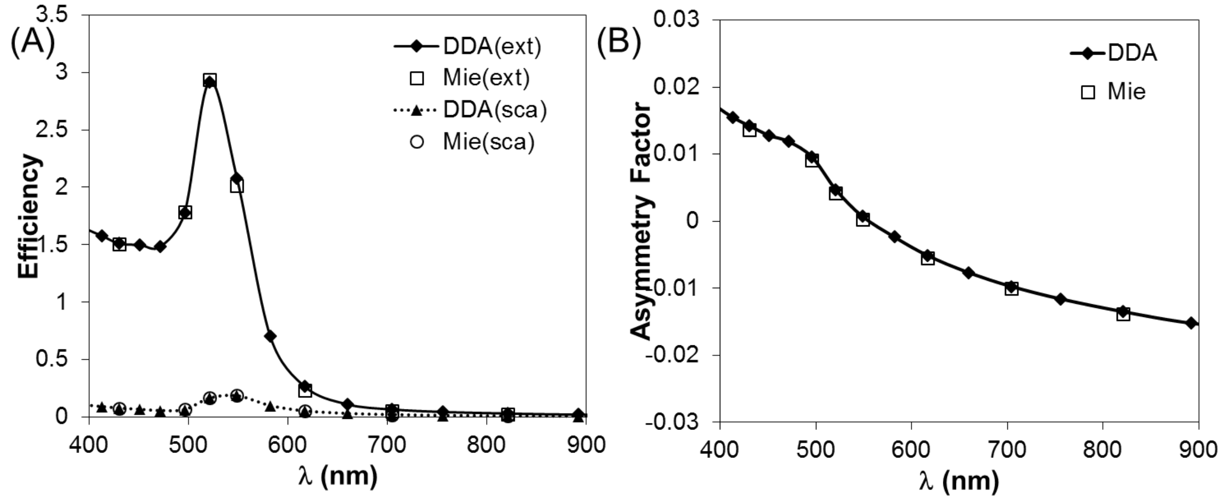

Figure S2. Validation of DDA simulation with Mie theory. (A) Agreement in extinction and scattering (thus absorption as well) efficiency factors for 30nm GNS; (B) Agreement in asymmetry factor for the same GNS. Other sizes of GNS were also compared with good agreement and not shown.

2. Photothermal heating experimental setup

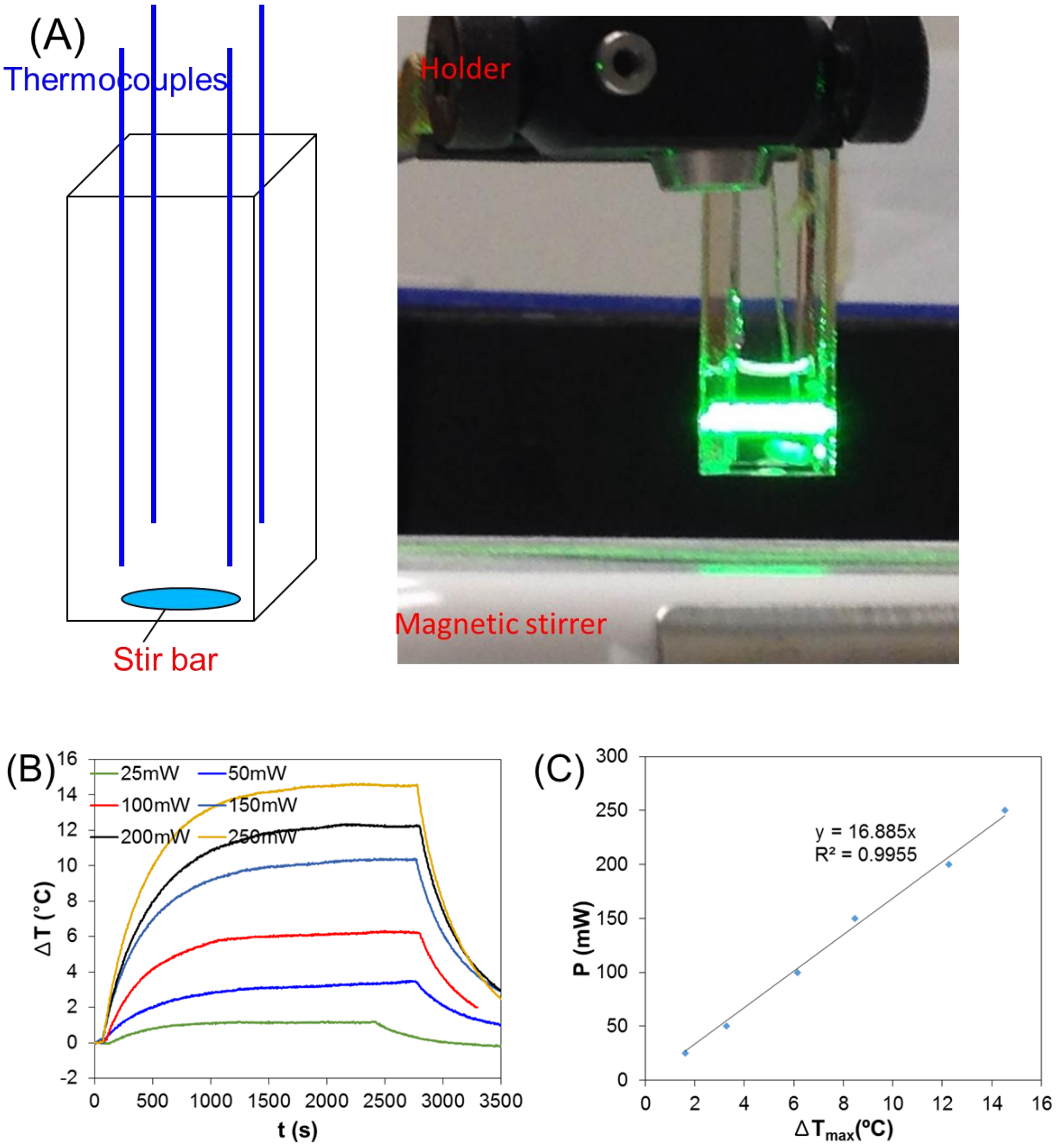

Figure S3. Photothermal heating experimental setup (A) Schematic and image of experimental setup; (B-C) Heating experiment calibration by resistor heating.

3. Measured GNR size distribution

Table S1. Length and diameter distribution binned into groups from TEM measurement (Figure 6 – Rod 1).

L_j \ D_i(nm)	6	8	10	12	Sum of columns, $f(L_j)$
**38**			1.56%	0.67%	2.2%
**34**		3.13%	3.79%	1.56%	8.5%
**32**		5.58%	4.91%		10.5%
**30**		9.15%	7.37%	0.22%	16.7%
**28**		10.94%	7.59%		18.5%
**26**		9.60%	3.13%		12.7%
**24**		8.71%	2.23%		10.9%
**22**	2.01%	4.46%	2.01%		8.5%
**20**		3.57%			3.6%
**18**	1.34%	2.68%			4.0%
**14**	2.01%	0.89%			2.9%
**10**	0.89%				0.9%
**Sum of rows, $f(D_i)$**	6.25%	58.71%	32.59%	2.46%	

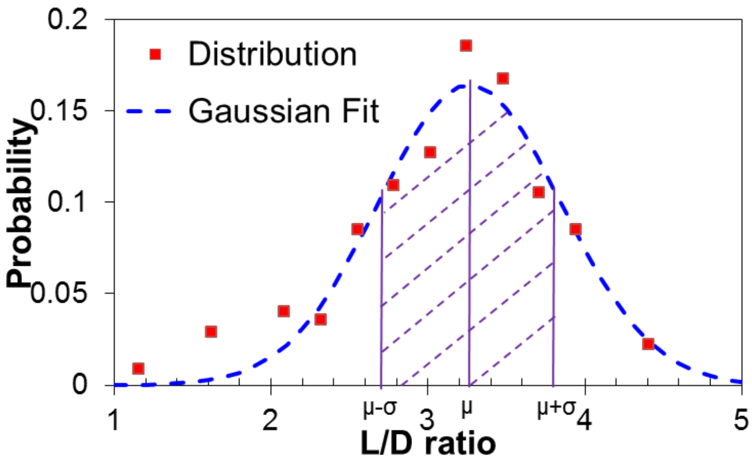

Figure S4. Gaussian fitting of GNR length/diameter ratio (L/D) distribution

The Gaussian fitting from Figure S2:

$$g(x) = \frac{1}{\sigma\sqrt{2\pi}} e^{-\frac{1}{2}\left(\frac{x-\mu}{\sigma}\right)^2}.$$

with fitted parameters: μ=3.3265; σ=0.583;

4. Comparison of DDA simulation and UV–Vis measurement for NIST particles.

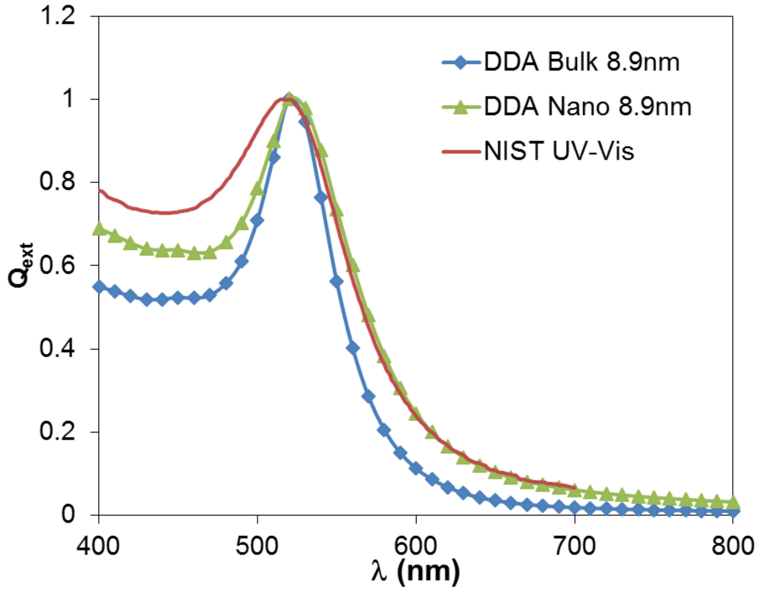

Figure S5. Comparison between prediction and measurement for 8.9nm GNP from NIST (RM 8011). DDA bulk refers to dielectric constants for bulk gold while DDA nano refers to size-corrected dielectric constants (as shown in Figure S1).

5. DDA Calculation protocol for GNR

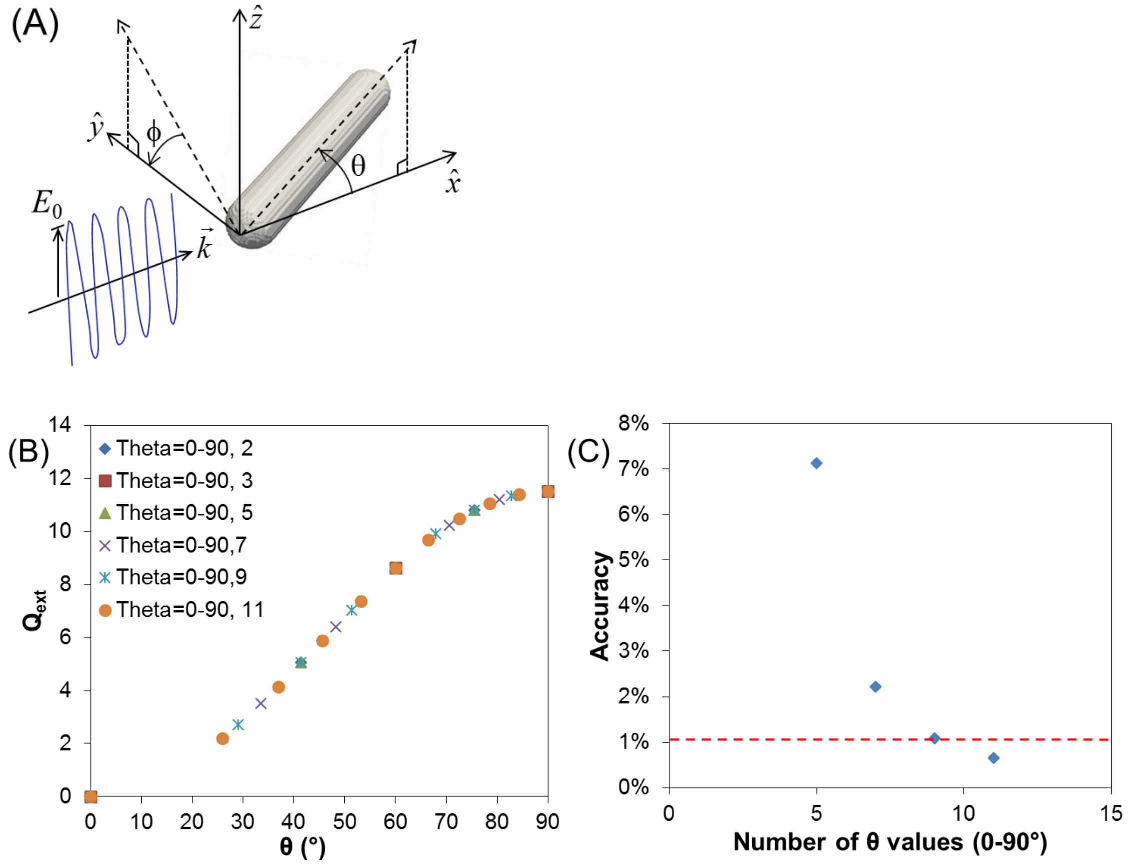

Figure S6. DDA calculation protocol. (A) Modeling of gold nanorod exposed to a plane electromagnetic wave of amplitude E_0 and wavevector $\vec{k}$ oriented along the $\hat{x}$-axis of the Cartesian reference. The rod orientation with respect to the Cartesian reference is characterized by the angle θ between the rod longitudinal axis and the *x*-axis and the angle ϕ between the rod short axis and the *y*-axis. (B-C) Systematically varying number of rotation angles (θ) shows that 9 rotation angles were required to obtain 1% accuracy.

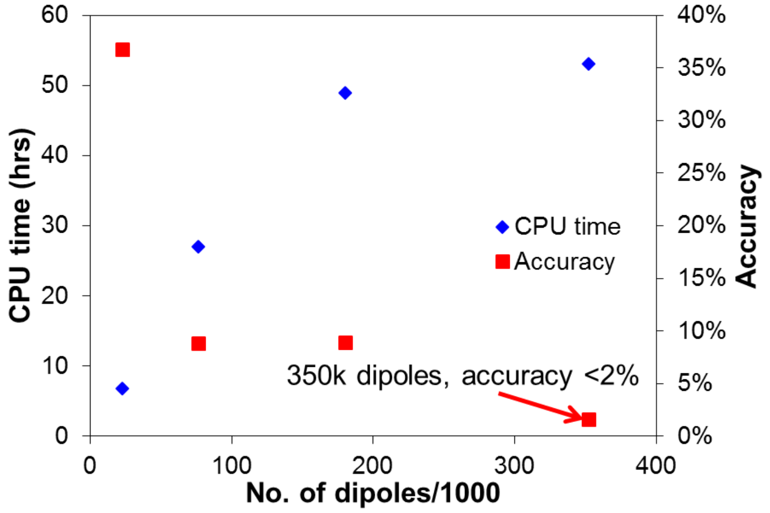

Figure S7. DDA calculation protocol establishment – CPU time and accuracy (i.e. convergence, defined as the percentage difference between two successive dipole densities) as a function of dipole number.

6. UV-Vis spectrophotometry for GNR before and after laser heating

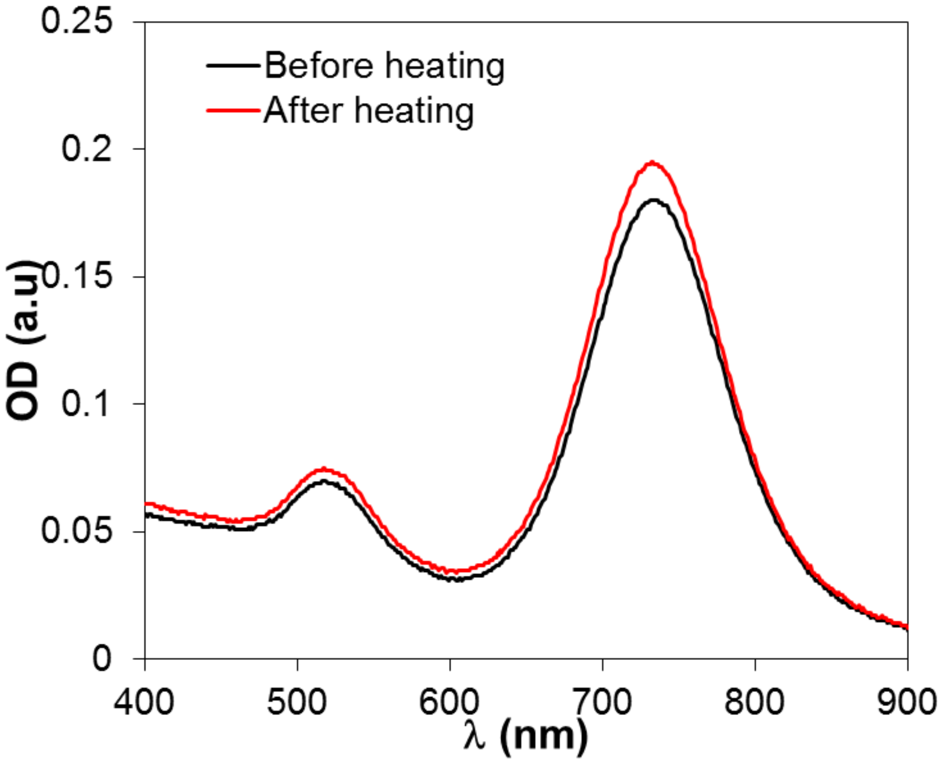

Figure S8. UV-Vis spectrophotometry for GNR before and after laser heating shows minimal peak shift. The small change in the magnitude of absorbance may be due to the water evaporation during heating, thus concentrating the GNR sample.
